# The effect of vitamin D supplementation on pain: an analysis of data from the D-Health randomised controlled trial

**DOI:** 10.1017/S0007114522003567

**Published:** 2023-08-28

**Authors:** Aninda Rahman, Mary Waterhouse, Catherine Baxter, Briony Duarte Romero, Donald S. A. McLeod, Bruce K. Armstrong, Peter R. Ebeling, Dallas R. English, Gunter Hartel, Michael G. Kimlin, Rachel O’Connell, Jolieke C. van der Pols, Alison J. Venn, Penelope M. Webb, David C. Whiteman, Rachel E. Neale

**Affiliations:** 1 Communicable Disease Control (CDC), Directorate General of Health Services, Bangladesh; 2 Population Health Department, QIMR Berghofer Medical Research Institute, Brisbane, 4006, Australia; 3 Department of Endocrinology and Diabetes, Royal Brisbane and Women’s Hospital, Brisbane, Australia; 4 School of Public Health, University of Sydney, Sydney, Australia; 5 Department of Medicine, School of Clinical Sciences, Monash University, Melbourne, Australia; 6 Melbourne School of Population Health, University of Melbourne, Melbourne, Australia; 7 Cancer Epidemiology Division, Cancer Council Victoria, Melbourne, Australia; 8 School of Biomedical Sciences, Queensland University of Technology, Australia; 9 NHMRC Clinical Trials Centre, University of Sydney, Sydney, Australia; 10 Queensland University of Technology (QUT), Faculty of Health, School of Exercise and Nutrition Sciences, Brisbane, Australia; 11 Menzies Institute for Medical Research, University of Tasmania, Hobart, Australia; 12 School of Public Health, The University of Queensland, Brisbane, Australia

**Keywords:** Vitamin D, Vitamin D_3_, Pain, Chronic pain, Bodily pain, PIQ-6, Randomised controlled trial

## Abstract

Observational studies suggest that 25-hydroxy vitamin D (25(OH)D) concentration is inversely associated with pain. However, findings from intervention trials are inconsistent. We assessed the effect of vitamin D supplementation on pain using data from a large, double-blind, population-based, placebo-controlled trial (the D-Health Trial). 21 315 participants (aged 60–84 years) were randomly assigned to a monthly dose of 60 000 IU vitamin D_3_ or matching placebo. Pain was measured using the six-item Pain Impact Questionnaire (PIQ-6), administered 1, 2 and 5 years after enrolment. We used regression models (linear for continuous PIQ-6 score and log-binomial for binary categorisations of the score, namely ‘some or more pain impact’ and ‘presence of any bodily pain’) to estimate the effect of vitamin D on pain. We included 20 423 participants who completed ≥1 PIQ-6. In blood samples collected from 3943 randomly selected participants (∼800 per year), the mean (sd) 25(OH)D concentrations were 77 (sd 25) and 115 (sd 30) nmol/l in the placebo and vitamin D groups, respectively. Most (76 %) participants were predicted to have 25(OH)D concentration >50 nmol/l at baseline. The mean PIQ-6 was similar in all surveys (∼50·4). The adjusted mean difference in PIQ-6 score (vitamin D cf placebo) was 0·02 (95 % CI (−0·20, 0·25)). The proportion of participants with some or more pain impact and with the presence of bodily pain was also similar between groups (both prevalence ratios 1·01, 95 % CI (0·99, 1·03)). In conclusion, supplementation with 60 000 IU of vitamin D_3_/month had negligible effect on bodily pain.

Vitamin D plays a vital role in calcium homeostasis and skeletal development. Observational studies suggest that vitamin D is associated with many other health conditions, and that it might also influence pain^(1)^. Pain is a common symptom in many diseases, and chronic or persistent pain unrelated to other disease processes is a major public health problem^(2)^. In a global burden of disease review, chronic pain was identified as the leading cause of disability^(3)^. It has high socio-economic impact; it decreases productivity and is estimated to cost 1·5 % to 3 % of European countries’ total gross domestic product^(4)^. In the 2012 USA National Health Interview Survey, 56 % of adults reported experiencing pain within the 3 months prior to the survey^(5)^. In a meta-analysis of population-based studies from the UK, the prevalence of chronic or persistent pain ranged from 35 % to 51 % (pooled estimate 44 %), with higher prevalence in adults aged 75 or above (pooled estimate 62 %)^(6)^. A study of 17 543 Australian adults reported the overall prevalence of chronic pain to be 17 % in men and 20 % in women, with chronic pain defined as pain experienced daily for 3 months^(7)^. Current interventions do not completely ameliorate chronic or persistent pain^(8)^ and, given the high prevalence, identifying alternative or adjunctive therapies is of interest.

Vitamin D may affect chronic pain through anatomic, hormonal, neurological and immunological pathways^(9)^. Systematic reviews and meta-analyses of observational studies have found significant associations between lower serum 25-hydroxy vitamin D (25(OH)D) concentrations and higher prevalence of pain^(1,10–12)^. However confounding or reverse causality is a possible explanation for these findings, with pain leading to reduced time outdoors and lower vitamin D status as a consequence. Randomised controlled trials (RCT) of vitamin D supplementation have shown inconsistent results. In three systematic reviews of RCT, vitamin D supplementation was not shown to reduce pain^(13–15)^. One systematic review and meta-analysis found that vitamin D supplementation resulted in a greater decrease in pain score compared with placebo, but most RCT were in hospital-based samples (*n* studies = 6, *n* participants = 456) and there was no effect in the community-based studies (*n* studies = 2, *n* participants = 2001)^(16)^. However, 96 % of participants in the community-based studies were from a trial that used a relatively low dose of vitamin D (400 IU of vitamin D/day) and that specifically measured joint pain^(17)^. The ViDA RCT (*n* 4055) from New Zealand found that monthly supplementation with 100 000 international units (IU) of vitamin D_3_ did not improve pain or reduce the need for analgesics^(18)^.

Given the inconsistent results from RCT, and the paucity of studies in the general population, we studied pain in a large, population-based RCT, the D-Health Trial. We aimed to assess the effect of monthly supplementation with 60 000 IU of vitamin D_3_ for up to 5 years on pain.

## Methods

### Study design, participants and intervention

The D-Health Trial is a randomised, double-blinded, placebo-controlled trial among Australian adults. Detailed methods have been described previously^(19)^. Between January 2014 and May 2015, 21 315 participants (aged 60–84 years) were randomly allocated (1:1 ratio) to take a monthly oral dose of 60 000 IU of vitamin D_3_ or a matching placebo for 5 years. The primary outcome of the D-Health Trial was all-cause mortality^(20)^. Pain is one of the tertiary outcomes^(19)^.

This study was conducted according to the guidelines laid down in the Declaration of Helsinki and all procedures involving human participants were approved by the QIMR Berghofer Medical Research Institute Human Research Ethics Committee (P1519). Written or online informed consent was obtained from all participants. It is registered with the Australian New Zealand Clinical Trials Registry (https://anzctr.org.au/): ACTRN12613000743763.

The Australian commonwealth electoral roll was used as the sampling frame. It is compulsory for all Australians aged 18 years and over to enrol to vote and 95 % of eligible people are enrolled^(21)^. We also sought volunteers through the media and from participants’ friends and families. People who reported a previous diagnosis of osteomalacia, hypercalcaemia, hyperparathyroidism, sarcoidosis or kidney stones, or who were taking more than 500 IU of supplementary vitamin D/d were not eligible.

We used automated computer-generated permuted block randomisation, stratified by age (60–64, 65–69, 70–74, 75+), sex and state of residence to assign participants to the vitamin D or placebo groups. Staff and investigators did not have access to the allocation list.

Vitamin D gel capsules contained 60 000 IU vitamin D_3_ in a soya oil excipient. Placebo gel capsules contained excipient only and were identical in appearance. We mailed participants twelve capsules at the beginning of the study, and annually thereafter.

### Baseline characteristics

Participants completed a survey prior to randomisation to capture demographic, lifestyle and health characteristics. We did not measure 25(OH)D concentration at baseline; rather we used a statistical model to predict 25(OH)D concentration at baseline, developed and validated using blood samples and data collected from a random sample of placebo group participants throughout the trial^(22)^.

### Assessment of pain

Pain was measured by the Pain Impact Questionnaire (PIQ-6) in surveys administered 1, 2, and 5 years after enrollment. The PIQ-6 is a validated self-administered brief questionnaire which asks about bodily pain and its impact on everyday activities in the last 4 weeks^(23,24)^. Responses to the PIQ-6 were used to calculate a score that can take values from 37 to 82, with a higher score reflecting greater pain impact. We did not calculate pain scores for participants who did not complete all items in the PIQ-6.

The PIQ-6 score, treated as a continuous variable, was the primary outcome in this analysis. We estimated, based on results from the ViDA Trial^(18)^, that we would have 80 % power to detect a difference of 0·33 in the mean PIQ-6 score between the two groups after 2 years of follow-up, allowing for 10 % loss-to-follow-up from baseline.

We included two secondary outcomes. Firstly, we categorised PIQ-6 scores into two groups: ‘little or no pain impact’ (PIQ-6 ≤ 50) and ‘some or more pain impact’ (PIQ-6 > 50)^(25)^. Secondly, we used responses to the first question of the PIQ-6 questionnaire (i.e. in the past 4 weeks, how much bodily pain have you had?) to categorise participants as having ‘no bodily pain’ (if the response was ‘none’ or ‘very mild’) and ‘some bodily pain’ (if the response was ‘mild’, ‘moderate’, ‘severe’ or ‘very severe’).

### Monitoring adherence and adverse events

Each year we measured serum 25(OH)D concentration in a random sample of participants, with selection stratified by study group, age group, sex, state and month of randomisation. A laboratory taking part in the international vitamin D standardisation program used a tandem liquid chromatography mass spectroscopy assay to measure 25(OH)D concentration^(26)^. In surveys administered each year, participants were asked to report the number of study tablets taken in the previous year and their use of off-trial vitamin D supplements. We asked participants to report all adverse events to the study helpline, and hypercalcaemia, hyperparathyroidism, sarcoidosis and kidney stones were also captured in annual surveys.

### Statistical analysis

We used SAS software version 9.4 for Windows (copyright © 2013, SAS Institute Inc.) and R version 4.0.3^(27)^ with the ggplot2 package^(28)^, and followed a pre-specified statistical analysis plan. Statistical code was developed using a dataset in which the randomisation group and participant identifier variables had been removed; participants were randomly assigned to two groups of equal size. After code was checked and verified, it was implemented on a data set containing the randomisation group variable. We use a significance level of *P* < 0·05 with no adjustment for multiple testing.

We analysed data using a modified intention-to-treat approach, including participants for whom at least one PIQ-6 score was available and analysing them in the groups to which they were assigned. We compared the distribution of baseline characteristics between participants who were included and excluded from the analysis using chi-squared tests.

We used linear regression to analyse PIQ-6 score as a continuous variable, and log-linked regression to estimate prevalence ratios (PR) for binary outcomes (i.e. some or more pain impact and some bodily pain). All models included the randomisation stratification variables of age, sex and state of residence. We analysed data from each survey separately and fitted a model integrating data from all surveys, using generalised estimating equations with an exchangeable correlation matrix to account for intra-person correlation. We included time (1, 2 or 5 years) in the generalised estimating equations models. To investigate whether the effect of supplementation varied with time since randomisation, we fitted an additional model that included a time by study group interaction term.

To assess whether the effect of vitamin D supplementation on PIQ-6 score was modified by baseline values of age, sex, BMI, history of chronic pain or predicted baseline 25(OH)D concentration, we incorporated an interaction term (separately for each covariable) in the generalised estimating equations models that did not include a time by study group interaction term. We used these models to generate stratum-specific effect estimates.

To confirm that variables previously shown to be associated with pain were evident in our study cohort, we estimated associations between baseline factors and each pain outcome using generalised estimating equations models (linear and log-linked). The study groups were combined for these analyses and models included potential confounders, identified using directed acyclic graphs.

## Results

### Participants

Of the 21 315 participants randomised, 5 requested that their details be deleted from the database. Eighty-four percent of participants reported taking ≥80 % of the study capsules up to the end of 5 years^(20)^. Across the course of the trial, we collected 4441 blood samples from 3943 participants. The mean 25(OH)D concentration was 77 (sd 25) and 115 (sd 30) nmol/l in the placebo and vitamin D groups, respectively. Most (76 %) of participants were predicted to be vitamin D replete (25(OH)D concentration >50 nmol/l) at baseline.

**Fig. 1.**
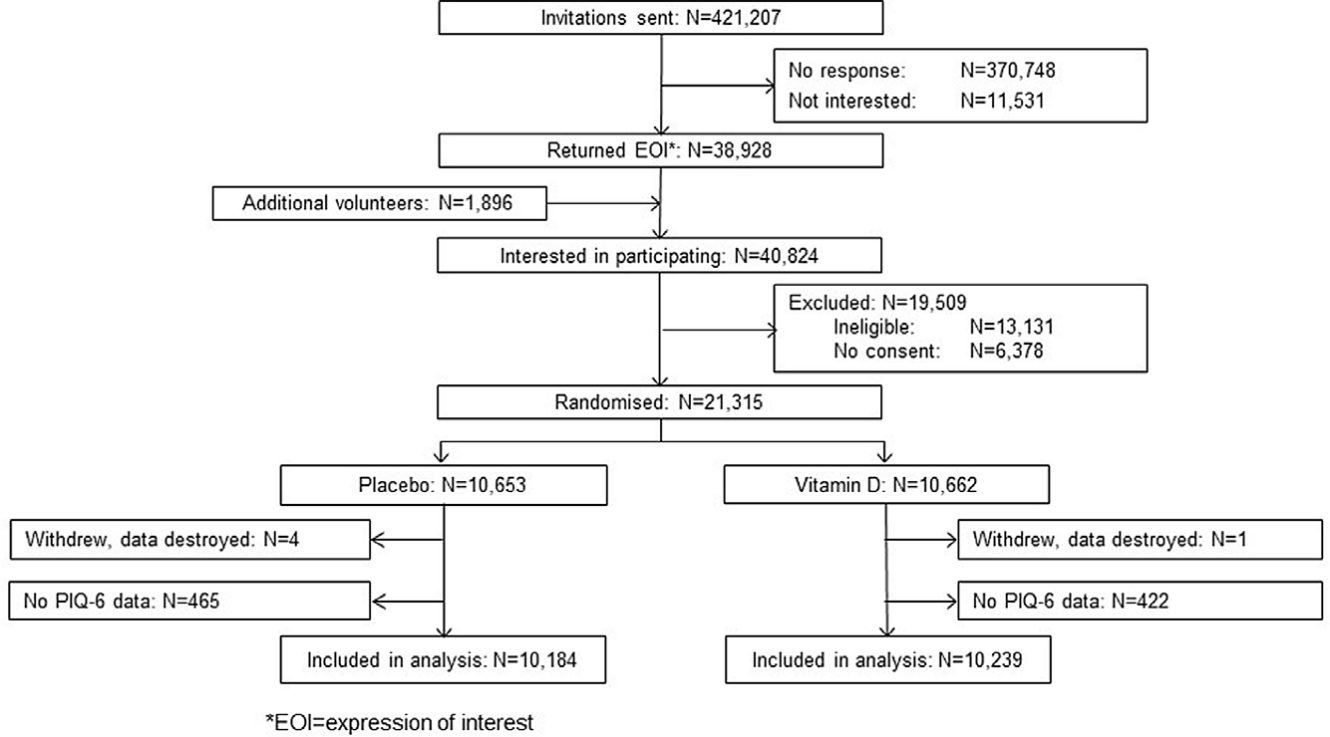
Flow chart of participant selection for analysis of pain.

For the current analysis, we included 20 423 participants (∼96 % of both study groups) for whom at least one PIQ-6 score was available (Fig. 1). The pattern of missing PIQ-6 data is presented in Supplementary Table 1. Participants who were not included in the analysis were more likely to be older and to have adverse lifestyle or health characteristics (online Supplementary Table 2). Placebo participants were slightly over-represented in the excluded group (online Supplementary Table 2) but the balance between the study groups was preserved in the included participants (Table 1). The mean PIQ-6 score in year 1 was slightly higher among participants for whom the PIQ-6 score was missing in year 5 than in those who completed the year 5 PIQ-6; this was the case in both study groups (online Supplementary Table 3).

**Table 1.** Baseline characteristics according to randomisation group

Characteristic	Vitamin D (*n* 10 239)		Placebo (*n* 10 184)	
	*n*	%	*n*	%
Sex				
Men	5533	54·0	5513	54·1
Women	4706	46·0	4671	45·9
Age (years)				
60–64	2558	25·0	2542	25·0
65–69	2822	27·6	2802	27·5
70–74	2779	27·1	2755	27·1
≥ 75	2080	20·3	2085	20·5
Predicted 25(OH)D concentration (nmol/l)				
< 50	2428	23·7	2459	24·1
≥ 50	7811	76·3	7725	75·9
BMI (kg/m^2^)				
< 25	3149	30·9	3021	29·8
25 to < 30	4296	42·1	4400	43·3
≥ 30	2762	27·1	2732	26·9
Missing	32		31	
Smoking history				
Never	5616	55·2	5558	55·0
Ex-smoker	4173	41·1	4103	40·6
Current	376	3·7	438	4·3
Missing	74		85	
History of diabetes				
No	9358	91·7	9336	91·9
Yes	843	8·3	822	8·1
Missing	38		26	
History of depression				
No	9648	94·6	9589	94·4
Yes	551	5·4	570	5·6
Missing	40		25	
History of chronic pain				
No	7988	78·3	7930	78·1
Yes	2211	21·7	2227	21·9
Missing	40		27	
History of arthritis				
No	5657	55·5	5555	54·8
Yes	4532	44·5	4587	45·2
Missing	50		42	
Living alone				
No	8186	80·3	8148	80·4
Yes	2004	19·7	1984	19·6
Missing	49		52	
Self-rated overall health				
Excellent, very good	5680	56·4	5612	55·9
Good, fair, poor	4395	43·6	4434	44·1
Missing	164		138	
Self-rated quality of life				
Excellent, very good	6762	67·5	6782	68·0
Good, fair, poor	3252	32·5	3192	32·0
Missing	225		210	

All the variables are self-reported other than sex, age and predicted 25(OH)D concentration.

25(OH)D, 25-hydroxy vitamin D.

### The effect of vitamin D supplementation on pain

The mean PIQ-6 score was similar across the three surveys (∼50). There was no effect of vitamin D supplementation on the PIQ-6 score at any survey point or overall (Table 2). The difference between the two groups was less than 0·2 points at all three time points; the overall effect estimate (vitamin D *cf* placebo) was 0·02 (95 % CI (0·20, 0·25)), and there was no evidence of an interaction between supplementation and time (*P* = 0·49).

**Table 2.** Effect of being randomised to vitamin D supplementation on PIQ-6 score, presence of some or more pain impact and presence of some bodily pain

	PIQ-6 score, mean	sd	Some or more pain impact*, *n*	%	Some bodily pain, *n*	%
Year 1						
Vitamin D (*n* 9733)	50·4	9·8	5008	51·5	4836	48·2
Placebo (*n* 9658)	50·4	9·7	4907	50·8	4751	47·6
Effect estimate (95 % CI)†,‡	0·04	−0·23, 0·31‖	1·01	0·99, 1·04¶	1·01	0·98, 1·04¶
Year 2						
Vitamin D (*n* 7630)	50·3	9·8	3893	51·0	3926	49·5
Placebo (*n* 7550)	50·4	9·9	3878	51·4	3864	49·1
Effect estimate (95 % CI)†,‡	−0·17	−0·48, 0·14‖	0·99	0·96, 1·02¶	1·01	0·98, 1·04¶
Year 5						
Vitamin D (*n* 9164)	49·9	8·9	3982	43·5	4255	46·2
Placebo (*n* 9029)	49·8	8·7	3840	42·5	4131	45·5
Effect estimate (95 % CI)†,‡	0·08	−0·17, 0·34‖	1·02	0·99, 1·06¶	1·01	0·98, 1·05¶
Overall effect estimate (95 % CI)§	0·02	−0·20, 0·25	1·01	0·99, 1·03	1·01	0·99, 1·03

PIQ, Pain Impact Questionnaire.

* Some or more pain impact: PIQ-6 score >50.

† Effect estimates comparing vitamin D with placebo (reference).

‡ Effect estimates adjusted for age, sex and state.

§ Effect estimate calculated using all available records; generalised estimating equations with exchangeable correlation matrix used to account for intra-person correlation.

‖ Adjusted mean difference (95 % CI) estimated using linear regression.

¶ Adjusted prevalence ratio (95 % CI) calculated using log-linked binomial regression.

Similarly, there were no significant differences between the vitamin D and placebo groups in the overall prevalence of some or more pain impact (PR 1·01; 95 % CI (0·99, 1·03)) or some bodily pain (PR 1·01; 95 % CI (0·99, 1·03)) (Table 2). There was no interaction with time (*P* = 0·32 and 0·94, respectively).

**Fig. 2.**
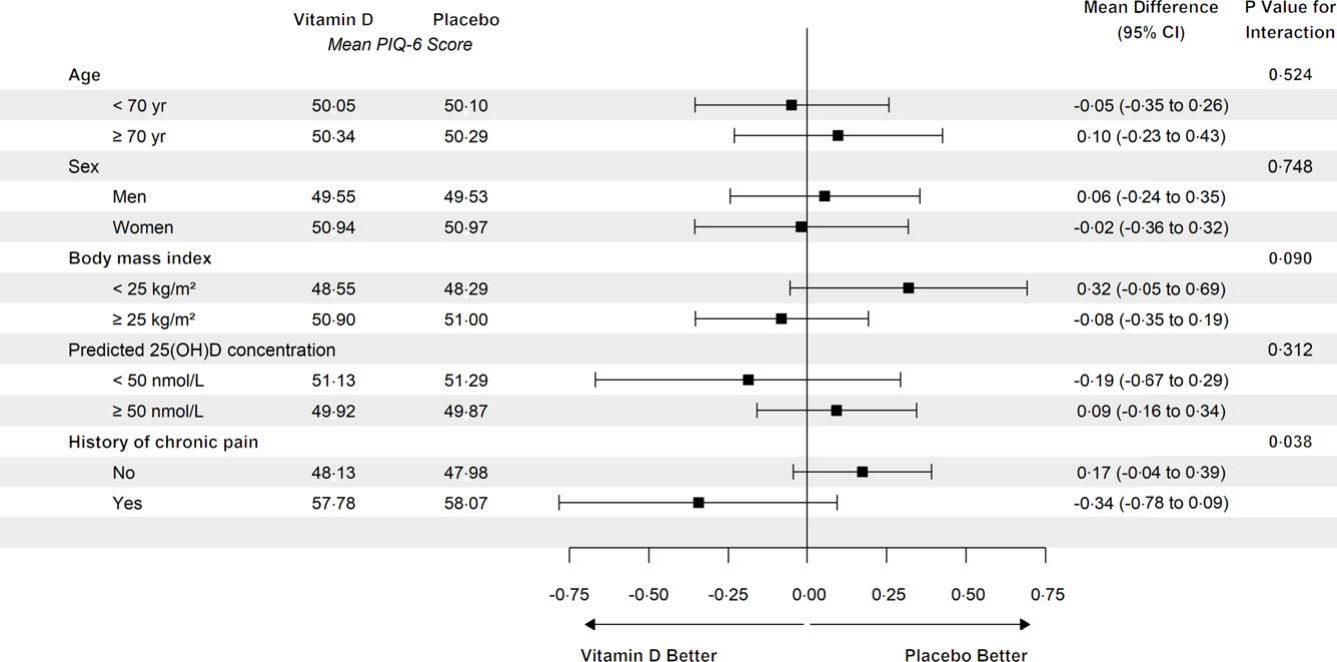
Effect of being randomised to vitamin D supplementation on mean pain score within subgroups. Here effect estimate (mean difference) is comparing vitamin D with placebo and it is adjusted for age, sex and state. Mean difference is calculated using all available records; generalised estimating equations with exchangeable correlation matrix are used to account for intra-person correlation. *P* value for interaction is from a linear model.

In the analysis of PIQ-6 score, there was a significant interaction between randomisation group and history of chronic pain at baseline (*P* = 0·038) but there was no evidence of benefit or harm in those with or without a history of pain (Fig. 2). There was no evidence of interaction with any other baseline variable examined (Fig. 2), and summary statistics for each pain outcome were similar between groups at each time (online Supplementary Table 4–6).

### Associations between baseline characteristics and pain

Mean PIQ-6 scores were significantly higher in participants aged ≥70 years *v*. those who were aged <70 years, women *v*. men, current or past smokers *v*. never smokers, participants with BMI ≥ 25 kg/m^2^ *v*. participants with BMI < 25 kg/m^2^, participants with predicted baseline serum 25(OH)D concentration <50 nmol/l *v*. participants with predicted concentration ≥50 nmol/l, and in those with diabetes, depression, high blood pressure or chronic pain compared with those without these conditions (online Supplementary Table 7). The strongest associations were with a history of chronic pain (adjusted mean PIQ-6 score difference 9·41; 95 % CI (9·16, 9·67)) and depression (adjusted mean PIQ-6 score difference 5·07; 95 % CI (4·52, 5·62)).

## Discussion

In this large RCT, supplementing older adults with 60 000 IU of vitamin D/month did not influence pain. This finding was consistent in all subgroups and at all time points.

This analysis has a number of strengths. It was nested in a large population-based RCT with a long intervention period, providing information to inform population-level interventions. The large monthly dose of vitamin D caused a substantial difference in 25(OH)D concentration between the study groups and adherence to the intervention was high. The lack of a baseline PIQ-6 measure is a limitation. However, all other characteristics, including a history of chronic pain, were well balanced at baseline, suggesting this would have also been the case for PIQ-6 scores. Further there was little difference in the percentage with missing PIQ-6 data between study groups, and the PIQ-6 score at year 1 was minimally associated with missing PIQ-6 at subsequent time points. Thus, the results are unlikely to be biased by differential withdrawal.

The prevalence of bodily pain among the participants included in this analysis was comparable to the Australian population. In the 2011–2012 Australian Health Survey approximately three-quarters of people aged 65–74 reported experiencing pain of any severity in the 4 weeks prior to interview^(29)^; this is very similar to the 74 % reported by D-Health participants in the same age group. The mean PIQ-6 score (50·4) was also similar to the value of 52·0 reported in 650 people aged 65–74 years recruited from the general population of the USA^(23)^.

The lack of effect of vitamin D supplementation on pain is consistent with the findings of a similar trial from New Zealand^(18)^, the ViDA Trial, in which 5110 older adults were supplemented with 100 000 IU/month for a median of 3·3 years. Among the 4055 participants included in the analysis of pain, there was no effect on PIQ-6 score, but among participants who were vitamin D deficient (25(OH)D < 50 nmol/l) at baseline vitamin D reduced use of non-steroidal anti-inflammatory drugs (adjusted risk ratio of 0·87, 95 % CI (0·78, 0·96)). In the absence of any other significant findings, chance would be a plausible explanation for that result.

Our null finding is also supported by several systematic reviews and meta-analyses^(13–15)^. Most reviews did not report any conclusive effect of vitamin D supplementation on pain. A recent meta-analysis did find a small but significant effect of vitamin D supplementation on the mean change in pain score from baseline to follow-up (mean difference = −0·57, 95 % CI (−1·00, −0·15))^(16)^. The effect was restricted to studies conducted in hospital-based settings, but the vast majority of participants included in the analysis of community-based studies were randomised to 400 IU of vitamin D, which may have been too low to elicit any effect.

The D-Health and ViDA Trials used monthly bolus doses of vitamin D. It is possible that bolus dosing confers less benefit than daily dosing, even though it results in a similar 25(OH)D concentration^(30)^. For some health outcomes, evidence is emerging to suggest that daily dosing delivers benefits but less frequent dosing does not. This is most notable for acute respiratory tract infection, although the difference between the dosing regimens was not statistically significant in the most recent meta-analysis^(31)^. There is insufficient evidence to compare the effect of daily *v*. bolus dosing on pain.

We used a questionnaire instrument that measures overall bodily pain and the impact of the pain. It is possible that vitamin D has effects on pain with specific underlying causes, such as fibromyalgia, which may be under-represented in the study population. However, the strong associations with known risk factors suggest that the PIQ-6 is a robust measure of overall pain, and it is reasonable to conclude that there is no impact on pain arising from the most common causes in older people (i.e. osteoarthritis, rheumatoid arthritis, osteoporosis)^(32)^.

Vitamin D supplementation may only be of benefit for pain in people who are vitamin D deficient, but this has not been adequately explored. One RCT found significant improvement of low back pain only in patients with serum 25(OH)D concentration <30 nmol/l, after a bolus oral dose of 100 000 IU followed by 4000 IU/day of vitamin D supplementation, but in the ViDA Trial there was no evidence of benefit in participants with serum 25(OH)D concentration < 50 nmol/l^(18)^. We found no interaction with predicted baseline serum 25(OH)D concentration, but this may have been due to the relatively low positive predictive value of our model^(22)^; that is, it is likely that a relatively high proportion of people classified as having low vitamin D status were actually vitamin D sufficient.

The mean concentration of serum 25(OH)D in our study population was somewhat higher than in the Australian general population^(33)^ (77 nmol/l *v*. 69 nmol/l), but this may be at least partially due to differences in the geographic distribution of the study populations, time of year of sample collection or difference in analytic laboratories. Importantly, most participants were predicted to be vitamin D replete at baseline, possibly due to Australia’s relatively high ambient ultraviolet radiation; thus while our findings may be generalisable to populations that are largely vitamin D replete, they are uninformative about the potential benefit of treating clinical vitamin D deficiency.

In conclusion, this analysis of a large population-based trial suggests that vitamin D is unlikely to have any clinically relevant effect on non-specific pain in a population with a low prevalence of vitamin D deficiency. It is plausible that treating deficiency would have benefit for pain, but these findings suggest that population-level supplementation in settings where the prevalence of vitamin D deficiency is low is not warranted.

## Supplementary material

For supplementary material accompanying this paper visit https://doi.org/10.1017/S0007114522003567. click here to view supplementary material
