# Supplementary material for: The effect of vitamin D supplementation on pain: an analysis of data from the D-Health randomised controlled trial
Source: Br J Nutr. 2022 Nov 25;130(4):633–40. doi: 10.1017/S0007114522003567 (PMC10357318; doi:10.1017/S0007114522003567)
Supplement: Supplementary file 1 [file S0007114522003567sup001.docx]

*Supplementary Table 1. Pattern of missing PIQ-6 score in annual surveys*

| Annual survey 1 | Annual survey 2 | Annual survey 5 | N | % |
| --- | --- | --- | --- | --- |
| Available | Available | Available | 13275 | 62.3 |
| Available | Available | Not Available | 1172 | 5.5 |
| Available | Not Available | Available | 4015 | 18.8 |
| Available | Not Available | Not Available | 929 | 4.4 |
| Not Available | Available | Available | 604 | 2.8 |
| Not Available | Available | Not Available | 129 | 0.6 |
| Not Available | Not Available | Available | 299 | 1.4 |
| Not Available | Not Available | Not Available | 887^1^ | 4.2 |

^1^This subset of participants was excluded from the final analysis

Supplementary Table 2. Baseline characteristics of participants included versus excluded from the final analysis

|  | N (%) | |  |
| --- | --- | --- | --- |
| Characteristic | Included (N = 20,423) | Excluded^1^ (N = 887) | P-value^2^ |
| **Sex** |  |  |  |
| Men | 11046 (54.1) | 484 (54.6) | 0.78 |
| Women | 9377 (45.9) | 403 (45.4) |  |
| **Age (years)** |  |  |  |
| 60-64 | 5100 (25.0) | 152 (17.1) | 0.00 |
| 65-69 | 5624 (27.5) | 210 (23.7) |  |
| 70-74 | 5534 (27.1) | 262 (29.5) |  |
| ≥ 75 | 4165 (20.4) | 263 (29.7) |  |
| **Predicted 25(OH)D concentration (nmol/L)** |  |  |  |
| < 50 | 4887 (23.9) | 313 (35.3) | 0.00 |
| ≥ 50 | 15536 (76.1) | 574 (64.7) |  |
| **Body mass index (kg/m²)** |  |  |  |
| < 25 | 6170 (30.3) | 247 (29.7) | 0.11 |
| 25 to < 30 | 8696 (42.7) | 333 (40.1) |  |
| ≥ 30 | 5494 (27.0) | 251 (30.2) |  |
| *Missing* | *63* | *56* |  |
| **Smoking history** |  |  |  |
| Never | 11174 (55.1) | 418 (48.5) | 0.00 |
| Ex-smoker | 8276 (40.8) | 361 (41.9) |  |
| Current | 814 (4.0) | 82 (9.5) |  |
| *Missing* | *159* | *26* |  |
| **History of diabetes** |  |  |  |
| No | 18694 (91.8) | 767 (89.2) | 0.01 |
| Yes | 1665 (8.2) | 93 (10.8) |  |
| *Missing* | *64* | *27* |  |
| **History of depression** |  |  |  |
| No | 19237 (94.5) | 803 (93.4) | 0.16 |
| Yes | 1121 (5.5) | 57 (6.6) |  |
| *Missing* | *65* | *27* |  |
| **History of chronic pain** |  |  |  |
| No | 15918 (78.2) | 637 (74.1) | 0.00 |
| Yes | 4438 (21.8) | 223 (25.9) |  |
| *Missing* | *67* | *27* |  |
| **History of arthritis** |  |  |  |
| No | 11212 (55.1) | 483 (56.6) | 0.42 |
| Yes | 9119 (44.9) | 371 (43.4) |  |
| *Missing* | *92* | *33* |  |
| **Living alone** |  |  |  |
| No | 16334 (80.4) | 626 (71.9) | 0.00 |
| Yes | 3988 (19.6) | 245 (28.1) |  |
| *Missing* | *101* | *16* |  |
| **Self-rated overall health** |  |  |  |
| Excellent, Very good | 11292 (56.1) | 355 (42.1) | 0.00 |
| Good, Fair, Poor | 8829 (43.9) | 489 (57.9) |  |
| *Missing* | *302* | *43* |  |
| **Self-rated quality of life** |  |  |  |
| Excellent, Very good | 13544 (67.8) | 416 (50.3) | 0.00 |
| Good, Fair, Poor | 6444 (32.2) | 411 (49.7) |  |
| *Missing* | *435* | *60* |  |
| **Randomization group** |  |  |  |
| Placebo | 10184 (49.9) | 465 (52.4) | 0.14 |
| Vitamin D | 10239 (50.1) | 422 (47.6) |  |

^1^Participants with no PIQ-6 score at all time points were excluded from the final analysis.

^2^P value from chi-squared test

*All variables are self-reported other than sex, age and predicted 25(OH)D concentration*

Supplementary Table 3. PIQ-6 score at year 1 according to availability of PIQ-6 scores at years 2 and 5.

|  | | | Mean PIQ-6 score at Year 1 | |
| --- | --- | --- | --- | --- |
| PIQ-6 score at year 2 | PIQ-6 score at year 5 | N | Vitamin D | Placebo |
| Available | Available | 13275 | 50.2 | 50.2 |
| Available | Not Available | 1172 | 52.7 | 53.0 |
| Not Available | Available | 4015 | 50.2 | 50.0 |
| Not Available | Not Available | 929 | 52.1 | 51.4 |

Supplementary Table 4. Mean PIQ-6 score within subgroups according to treatment allocation at randomization

|  | **Year 1 mean (SD)** | | **Year 2 mean (SD)** | | **Year 5 mean (SD)** | |
| --- | --- | --- | --- | --- | --- | --- |
|  | **Vitamin D (N = 9733)** | **Placebo (N = 9658)** | **Vitamin D (N = 7630)** | **Placebo (N = 7550)** | **Vitamin D (N = 9164)** | **Placebo (N = 9029)** |
| **Sex** |  |  |  |  |  |  |
| Men | 49.8 (9.7) | 49.7 (9.7) | 49.7 (9.7) | 49.7 (9.7) | 49.2 (8.7) | 49.2 (8.4) |
| Women | 51.2 (9.8) | 51.2 (9.8) | 51.1 (9.8) | 51.4 (10.0) | 50.6 (9.0) | 50.4 (9.0) |
| **Age (years)** | | | | | | |
| < 70 | 50.3 (9.8) | 50.3 (9.7) | 50.2 (9.8) | 50.4 (9.7) | 49.6 (8.7) | 49.6 (8.6) |
| ≥ 70 | 50.5 (9.7) | 50.4 (9.8) | 50.4 (9.8) | 50.5 (10.0) | 50.1 (9.0) | 50.0 (8.9) |
| **Body mass index (kg/m²)** | | | | | | |
| < 25 | 48.8 (9.3) | 48.5 (9.0) | 48.8 (9.3) | 48.7 (9.2) | 48.1 (8.0) | 47.8 (7.8) |
| ≥ 25 | 51.1 (9.9) | 51.2 (9.9) | 50.9 (9.9) | 51.2 (10.0) | 50.7 (9.1) | 50.6 (9.0) |
| **Predicted 25(OH)D concentration (nmol/L)** | | | | | | |
| < 50 | 51.2 (10.0) | 51.4 (10.1) | 51.4 (10.0) | 51.7 (10.5) | 50.9 (9.2) | 50.8 (9.4) |
| ≥ 50 | 50.2 (9.7) | 50.0 (9.6) | 50.0 (9.7) | 50.1 (9.7) | 49.6 (8.7) | 49.4 (8.5) |
| **History of chronic pain** | | | | | | |
| No | 48.2 (8.8) | 47.9 (8.6) | 48.2 (9.0) | 48.2 (8.9) | 48.0 (7.9) | 47.9 (7.7) |
| Yes | 58.6 (8.5) | 58.9 (8.5) | 58.0 (8.7) | 58.4 (8.9) | 56.7 (8.8) | 56.8 (8.7) |

Supplementary Table 5. Prevalence of ‘some or more pain’ within subgroups according to treatment allocation at randomization

|  | **N (%) of participants experiencing some or more pain impact^1^** | | | | | |
| --- | --- | --- | --- | --- | --- | --- |
|  | **Year 1** | | **Year 2** | | **Year 5** | |
|  | **Vitamin D (N = 9733)** | **Placebo (N = 9658)** | **Vitamin D (N = 7630)** | **Placebo (N = 7550)** | **Vitamin D (N = 9164)** | **Placebo (N = 9029)** |
| **Sex** |  |  |  |  |  |  |
| Men | 2568 (48.6) | 2539 (48.4) | 2028 (48.6) | 2022 (48.6) | 1972 (40.4) | 1947 (40.2) |
| Women | 2440 (54.9) | 2368 (53.6) | 1865 (53.9) | 1856 (54.7) | 2010 (46.9) | 1893 (45.3) |
| **Age (years)** | | | | | | |
| < 70 | 2649 (51.6) | 2581 (50.7) | 2041 (50.8) | 2044 (51.4) | 2095 (42.3) | 2049 (41.8) |
| ≥ 70 | 2359 (51.3) | 2326 (51.0) | 1852 (51.3) | 1834 (51.4) | 1887 (44.9) | 1791 (43.4) |
| **Body mass index (kg/m²)** | | | | | | |
| < 25 | 1337 (44.8) | 1224 (42.7) | 1047 (44.1) | 982 (44.2) | 1009 (35.6) | 899 (32.9) |
| ≥ 25 | 3656 (54.4) | 3672 (54.3) | 2830 (54.0) | 2885 (54.4) | 2959 (46.9) | 2938 (46.8) |
| **Predicted 25(OH)D concentration (nmol/L)** | | | | | | |
| < 50 | 1271 (55.3) | 1264 (54.4) | 784 (56.5) | 825 (56.5) | 1015 (48.0) | 1012 (48.1) |
| ≥ 50 | 3737 (50.3) | 3643 (49.7) | 3109 (49.8) | 3053 (50.1) | 2967 (42.1) | 2828 (40.8) |
| **History of chronic pain** | | | | | | |
| No | 3204 (42.2) | 3077 (41.0) | 2547 (42.5) | 2462 (42.0) | 2522 (35.0) | 2393 (33.7) |
| Yes | 1791 (85.3) | 1817 (85.3) | 1332 (82.8) | 1404 (84.3) | 1445 (74.9) | 1433 (75.5) |

^1^Some or more pain impact: PIQ-6 score >50;

Supplementary Table 6. Prevalence of bodily pain within subgroups according to treatment allocation at randomization

|  | **N (%) of participants experiencing some or more bodily pain** | | | | | |
| --- | --- | --- | --- | --- | --- | --- |
|  | **Year 1** | | **Year 2** | | **Year 5** | |
|  | **Vitamin D (N = 9733)** | **Placebo (N = 9658)** | **Vitamin D (N = 7630)** | **Placebo (N = 7550)** | **Vitamin D (N = 9164)** | **Placebo (N = 9029)** |
| **Sex** |  |  |  |  |  |  |
| Men | 2446 (45.0) | 2387 (44.1) | 2027 (46.7) | 1983 (45.7) | 2099 (42.7) | 2059 (42.3) |
| Women | 2390 (52.0) | 2364 (51.8) | 1899 (52.8) | 1881 (53.4) | 2156 (50.1) | 2072 (49.2) |
| **Age (years)** | | | | | | |
| < 70 | 2502 (47.5) | 2428 (46.4) | 2004 (48.1) | 2008 (48.7) | 2238 (44.9) | 2193 (44.5) |
| ≥ 70 | 2334 (49.0) | 2323 (49.0) | 1922 (50.9) | 1856 (49.7) | 2017 (47.6) | 1938 (46.7) |
| **Body mass index (kg/m²)** | | | | | | |
| < 25 | 1291 (41.8) | 1206 (40.7) | 1051 (42.4) | 991 (42.5) | 1092 (38.3) | 1021 (37.2) |
| ≥ 25 | 3530 (51.0) | 3533 (50.6) | 2860 (52.6) | 2863 (52.0) | 3150 (49.7) | 3104 (49.1) |
| **Predicted 25(OH)D concentration (nmol/L)** | | | | | | |
| < 50 | 1217 (51.5) | 1239 (51.5) | 785 (54.1) | 835 (54.8) | 1084 (50.8) | 1065 (50.2) |
| ≥ 50 | 3619 (47.2) | 3512 (46.4) | 3141 (48.4) | 3029 (47.8) | 3171 (44.8) | 3066 (44.1) |
| **History of chronic pain** | | | | | | |
| No | 3024 (38.6) | 2880 (37.1) | 2529 (40.5) | 2411 (39.3) | 2693 (37.2) | 2601 (36.4) |
| Yes | 1799 (83.6) | 1856 (85.2) | 1383 (82.8) | 1443 (84.2) | 1550 (79.8) | 1516 (79.5) |

Supplementary Table 7. Association between potential risk factors and pain outcomes

| Risk factor | PIQ-6 score mean difference^1,2^ (95% CI) | Some or more pain impact risk ratio^1,2^ (95% CI) | Presence of bodily pain risk ratio^1,2^ (95% CI) | Covariates included in model |
| --- | --- | --- | --- | --- |
| Sex (female vs male) | 1.48 (1.24 to 1.71) | 1.12 (1.09 to 1.16) | 1.19 (1.15 to 1.22) | Age |
| Age (≥ 70 vs < 70 years) | 0.40 (0.16 to 0.63) | 1.02 (0.99 to 1.05) | 1.07 (1.04 to 1.11) | Sex |
| BMI (≥ 25 vs < 25 kg/m²) | 2.32 (2.08 to 2.57) | 1.23 (1.19 to 1.27) | 1.22 (1.18 to 1.26) | Sex, age, alcohol consumption, physical activity, education |
| Chronic pain (yes vs no) | 9.41 (9.16 to 9.67) | 1.98 (1.93 to 2.03) | 2.13 (2.08 to 2.19) | Sex, age, hypertension, obesity, education |
| Smoking (ex vs never) | 1.49 (1.25 to 1.74) | 1.13 (1.10 to 1.17) | 1.16 (1.13 to 1.20) | Sex, age, alcohol consumption, education |
| Smoking (current vs never) | 2.12 (1.43 to 2.82) | 1.14 (1.06 to 1.22) | 1.16 (1.08 to 1.25) | Sex, age, alcohol consumption, education |
| Predicted 25(OH)D concentration (< 50 vs ≥ 50 nmol/L) | 0.68 (0.38 to 0.98) | 1.05 (1.01 to 1.09) | 1.05 (1.02 to 1.09) | Sex, age, physical activity, obesity |
| Diabetes (yes v no) | 1.92 (1.43 to 2.40) | 1.14 (1.09 to 1.19) | 1.14 (1.09 to 1.20) | Sex, alcohol consumption, obesity |
| Hypertension (yes vs no) | 1.44 (1.20 to 1.68) | 1.12 (1.09 to 1.15) | 1.14 (1.10 to 1.17) | Sex, age, alcohol consumption, physical activity, education, obesity, smoking status |
| Depression (yes vs no) | 5.07 (4.52 to 5.62) | 1.36 (1.30 to 1.42) | 1.38 (1.32 to 1.44) | Sex, age, alcohol consumption, education, obesity, diabetes |

^1^ Effect estimates calculated using all available records; generalized estimating equations with exchangeable correlation matrix used to account for intra-person correlation

^2^ All covariates were categorical

CI=confidence interval
